# Isolated Murine Brain Model for Large-Scale Optoacoustic Calcium Imaging

**DOI:** 10.3389/fnins.2019.00290

**Published:** 2019-04-24

**Authors:** Sven Gottschalk, Oleksiy Degtyaruk, Benedict Mc Larney, Johannes Rebling, Xosé Luis Deán-Ben, Shy Shoham, Daniel Razansky

**Affiliations:** ^1^Institute for Biological and Medical Imaging, Helmholtz Center Munich, Neuherberg, Germany; ^2^Faculty of Medicine, Technical University of Munich, Munich, Germany; ^3^Faculty of Medicine, Institute of Pharmacology and Toxicology, University of Zurich, Zurich, Switzerland; ^4^Institute for Biomedical Engineering and Department of Information Technology and Electrical Engineering, ETH Zurich, Zurich, Switzerland; ^5^Tech4Health and Neuroscience Institutes and Department of Ophthalmology, New York University Langone Health, New York, NY, United States

**Keywords:** isolated brain, calcium dynamics, optoacoustic neuroimaging, functional neuroimaging, GCaMP6f

## Abstract

Real-time visualization of large-scale neural dynamics in whole mammalian brains is hindered with existing neuroimaging methods having limited capacity when it comes to imaging large tissue volumes at high speeds. Optoacoustic imaging has been shown to be capable of real-time three-dimensional imaging of multiple cerebral hemodynamic parameters in rodents. However, optoacoustic imaging of calcium activity deep within the mammalian brain is hampered by strong blood absorption in the visible light spectrum as well as a lack of activity labels excitable in the near-infrared window. We have developed and validated an isolated whole mouse brain preparation labeled with genetically encoded calcium indicator GCaMP6f, which can closely resemble *in vivo* conditions. An optoacoustic imaging system coupled to a superfusion system was further designed and used for rapid volumetric monitoring of stimulus-evoked calcium dynamics in the brain. These new imaging setup and isolated preparation’s protocols and characteristics are described here in detail. Our new technique captures calcium fluxes as true three-dimensional information across the entire brain with temporal resolution of 10 ms and spatial resolution of 150 μm, thus enabling large-scale neural recording at penetration depths and spatio-temporal resolution scales not covered with any existing neuroimaging techniques.

## Introduction

Each neuron’s output in a mammalian brain can be connected to up to 10,000 other neurons, relaying signals between each other via as many as 10^15^ synaptic connections (Azevedo et al., 2009). These highly interconnected basic working units of the brain form specialized neuronal sub-circuits, which functionally connect into a larger network, defining the overall brain architecture (Carpenter and Sutin, 1983). Neuronal activity occurs simultaneously and in a highly coordinated fashion in many different areas across the brain, serving as a fundamental representation of information processing and transmission within the nervous system. Understanding the brain’s circuit and network activity and to spatio-temporally map patterns of neuronal activity across large neuronal populations, distributed over the entire brain, is one of the most fundamental goals of neuroscience. For this, a number of approaches are being explored towards developing imaging systems that allow non-invasive observations of larger neuronal networks with high spatio-temporal resolutions, deep inside the brain.

Blood oxygen-level dependent functional magnetic resonance imaging is one of such functional neuroimaging techniques that detects blood flow and blood oxygenation changes in the brain (Huettel et al., 2004). Such cerebral hemodynamic changes are closely linked to neural activity via neurovascular coupling, (Logothetis et al., 2001) hence fMRI can be used to deduce observations of brain activity. This, however, is also the biggest caveat of fMRI as it can only indirectly assess neuronal activity. Electroencephalography (EEG) is another established method and a valuable tool for research and diagnosis, measuring voltage fluctuations resulting from ionic currents within neurons (Niedermeyer and da Silva, 2005). Non-invasive EEG allows recording of neuronal activity using electrodes placed along the scalp, and boasts millisecond-range temporal resolution (Hämäläinen et al., 1993). A very poor spatial resolution is, however, the critical limitation of EEG, and it can only record signals generated in the superficial layers of the cortex, while neuronal activity from deeper brain areas has far less contributions to the EEG signal (Srinivasan, 1999). A more recently published study also presented a paradigm for pure ultrasound imaging able to visualize stimulus-evoked brain activation in the somatosensory cortex of the rat brain with high spatiotemporal resolution, while also recording data on cerebral blood volume and flow (Macé et al., 2011). The need for an invasive approach using a cranial window and its inability to capture differences in blood oxygenation, however, limit the utility of this ultrasound-based method.

A variety of optical imaging methods that emerged during the last two decades are also becoming alternative approaches to observe the activity of large, distributed neuronal populations (Devor et al., 2012; Adesnik et al., 2014). Among them, macroscopic optical imaging techniques like diffuse optical tomography or near-infrared spectroscopy enable the monitoring of cerebral hemodynamics and measurements of cytochrome redox states across large neuronal populations and whole brains (Durduran et al., 2010). However, these methods again lack the necessary spatio-temporal resolution required for detailed analysis of fast-paced neuronal events. Furthermore, strong light scattering in the brain prevents deep brain imaging and thus only superficial regions can be examined using pure optical methods, preventing observations of deep neural activity (Devor et al., 2012; Liao et al., 2013).

Optoacoustic imaging methods have now emerged as a powerful alternative approach for imaging optical absorption in tissues and in the brain in particular. By combining the benefits of both ultrasound and optical imaging, the optoacoustic approach overcomes the limitations of pure optical methods, allowing for very specific, non-invasive molecular imaging up to several centimeters deep in tissue, picking up at depths where state-of-the-art optical microscopy techniques fail to penetrate (Dean-Ben et al., 2017). Novel optoacoustic imaging systems now also allow the non-invasive acquisition, processing and visualization of five-dimensional optoacoustic data (Dean-Ben and Razansky, 2014; Gottschalk et al., 2017). Functional optoacoustic brain imaging has been shown to deliver real-time, volumetric and spectrally enriched tomography recordings, (Gottschalk et al., 2015b) offering unique imaging performance in comparison to other neuroimaging modalities. Until recently, optoacoustic brain imaging focused on blood oxygenation variations and hemodynamics due to the strong and spectrally distinctive optoacoustic contrast provided by oxygenated and deoxygenated hemoglobin (Yao et al., 2014). The strong intrinsic contrast provided by blood has allowed for label-free visualizations of tissue hemodynamics, (Gottschalk et al., 2015b) stimulus-induced brain function (Liao et al., 2012) and seizure activity in mice (Tsytsarev et al., 2013; Gottschalk et al., 2017). Yet, hemodynamic changes only indirectly reflect neuronal activity.

Genetically encoded calcium indicators (GECIs) that modulate their fluorescence intensity as a function of intracellular calcium concentrations are potent tools for the direct observation of rapid activity in large neuronal networks. Functional optoacoustic neuro-tomography (FONT) has recently been shown capable of imaging fast calcium activity in zebrafish brains labeled with the GCaMP-family of GECI proteins (Deán-Ben et al., 2016). However, GCaMP excitation is usually done at 488 nm wavelength, which cannot be used for imaging deep inside living mammalian brains due to the high absorption by blood and limited light penetration at this wavelength.

In order to demonstrate the fundamental capacity for calcium imaging in whole rodent brains using FONT, we developed and validated an isolated-brain preparation from GCaMP6f-expressing mice and a custom imaging setup continuously perfused with artificial cerebrospinal fluid (ACSF). The model is subsequently shown to closely resemble *in vivo* conditions, exhibiting high viability and functional activity for several hours, while also preserving the indicator responses and a realistic optical light scattering environment. Once cleared from highly absorbing blood background, large-scale optoacoustic neural recording from mouse brains is enabled at penetration depths and spatio-temporal resolution scales not covered with the existing neuroimaging techniques.

## Results

### Validation of Brain Viability

**Figure 1:**
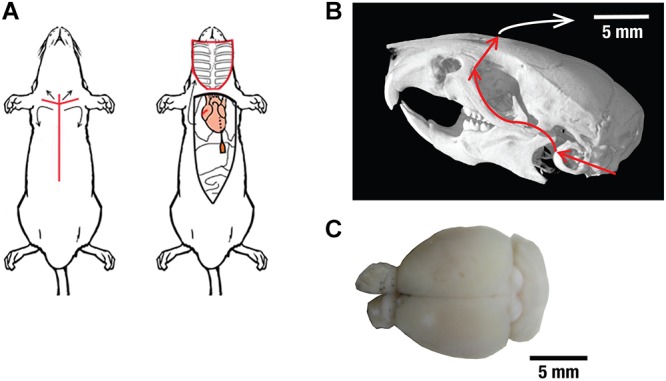
The brain extraction procedure. **(A)** After euthanasia, the surgery began with an incision from the mid abdomen to the sternum (left image). The ventral part of the ribcage was removed to allow unhindered access to the heart. Intracardiac perfusion was then carried out by insertion of a 25G butterfly needle into the left ventricle of the heart followed by an incision to the right atrium. Images are used under the Creative Commons Attribution 3.0 International License from reference (Gage et al., 2012). Incision markers, intestinal outlines and red color around the ribcage were added and images were cropped. **(B)** After decapitation, all tissue and skin surrounding the skull was removed and the skull was rinsed with ice-cold PBS to remove any remaining debris. Using a bone scissor, the skull plate was removed after cutting along the indicated red lines. Skull image from www.digimorph.org adapted with permission from T.B. Rowe. **(C)** Color photograph of an extracted brain after it has been separated from the skull.

**Figure 2:**
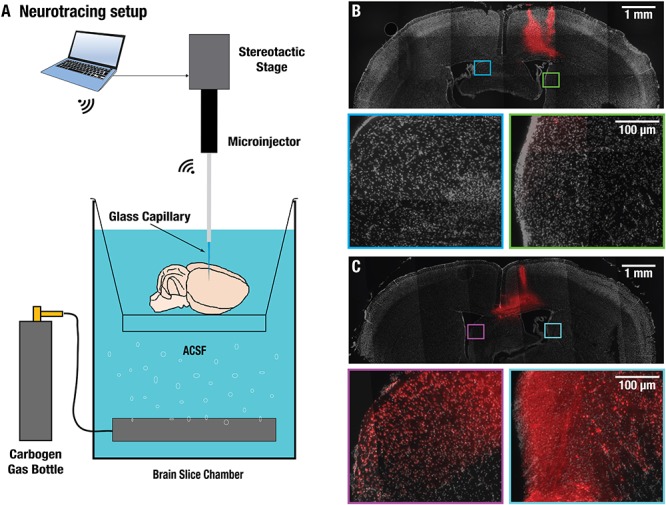
Validation of neural transport in excised brains. **(A)** Experimental setup used for injecting dextran neural tracer coupled to Texas-red directly into the cortex of excised brains under artificial cerebrospinal fluid (ACSF) and oxygen perfusion. For intra-brain injection a wireless robotic injection system with glass micro-capillary was utilized. **(B)** Compound microscopic images of a 50 μm-thick coronal brain slice fixated immediately after the injection showed no neuronal transport past the injection site. No labeled neurons could be detected in the square labeled areas. **(C)** Slice from a brain fixated after 1 h incubation time post-injection revealed structures stained by the tracer up to 2 mm away from the injection site, thus clearly indicating active transport of the tracer molecules in the neurons.

Swift extraction of an undamaged brain was vital for the viability of the isolated brains. The procedure was performed at 4°C and it took less than 10 min from the beginning of the intracardiac perfusion until commencement of the imaging experiments (Figure 1). Neuronal functionality of the isolated brains was first evaluated by intracortically injecting 0.5 μl of 10 kD dextran coupled with Texas red. This is a commonly used neuronal tracer that allows for assessment of both anterograde and retrograde transport and can only be transported by living neurons via a specialized channel (Schmued et al., 1990). The injection was carried out in the cortical area of excised CD-1 mouse brains (Figure 2). Clear signs of neuronal uptake in and around the injection site were detected with the Texas red fluorescence. As a negative control, Texas red without dextran was injected in another excised CD-1 mouse brain, and no signs of uptake on the cellular level were observed (data not shown). Brains fixated immediately after the injection showed no neuronal transport past the injection site (Figure 2B), while those fixated with paraformaldehyde after 1 h of incubation time post-injection revealed structures stained by the tracer up to 2 mm away from the injection site (Figure 2C), thus clearly indicating transport of the tracer molecules in the neurons. Notably, the injection location cannot precisely be selected since the commonly used reference point Bregma is no longer available in the isolated brains. However, similar labeling patterns were observed when comparing our results with tracer data from the publicly available mouse connectivity Allen Brain Atlas^1^.

**Figure 3:**
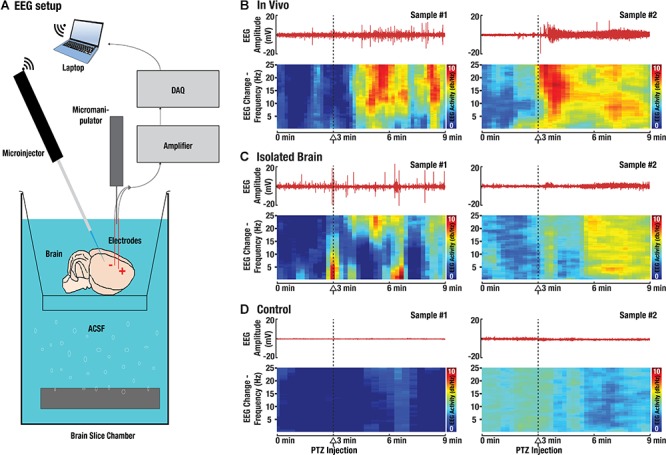
Validation of the isolated brain viability with electroencephalography (EEG) recordings. **(A)** The EEG activity was measured using two custom-made needle electrodes connected to a differential amplifier. In order to induce widespread brain activity, an epileptic agent pentylenetetrazol (PTZ) was injected intraperitoneally. **(B)** EEG signals recorded from mouse brains *in vivo* during injection of PTZ. The signal power spectra represent the frequency distribution of the brain activity. **(C)** The corresponding EEG responses recorded from isolated brains. **(D)** No EEG activity could be detected in negative controls, i.e., brains that were kept in PBS for 1 h to ensure cell death.

To further evaluate the functionality of the neurons in the isolated brain preparation, EEG-recordings during application of the epileptic drug pentylenetetrazol (PTZ) were carried out (Figure 3). For this, EEG-data was acquired from the cortex of isolated CD-1 mouse brains and compared to *in vivo* recordings from the cortex of an anesthetized CD-1 mouse. The excised brains were either placed in oxygenated ACSF and immediately measured, or kept in PBS for 1h to ensure brain death and serve as a negative control. Figure 3B–D show the recorded EEG signals along with their power spectrum representing the frequency distribution of the brain activity. Low frequency signal variations of 3–7 Hz were most prominent during baseline recordings, corresponding to the anesthetized state of the brain. The noise levels of the EEG-recordings in isolated brains were higher than those in the *in vivo* experiments. The additional background noise can be ascribed to the presence of bubbles produced during oxygenation of the ACSF-solution as well as the necessity of placing a grounding electrode inside the isolated brain. Following a baseline recording, neuronal stimulation was induced by PTZ injection (100 μl into the tail vein for the *in vivo* recording and 5 μl of PTZ intracortically for the excised brains). The EEG signal amplitude visibly increased and higher frequency signals in the range of 10–20 Hz appeared in the frequency distribution in both *in vivo* (Figure 3B) and isolated brains (Figure 3C), while no change could be detected in the negative control (Figure 3D). Overall, activity in the EEG-data lasted for up to 30 min in the isolated brain preparations.

**Figure 4:**
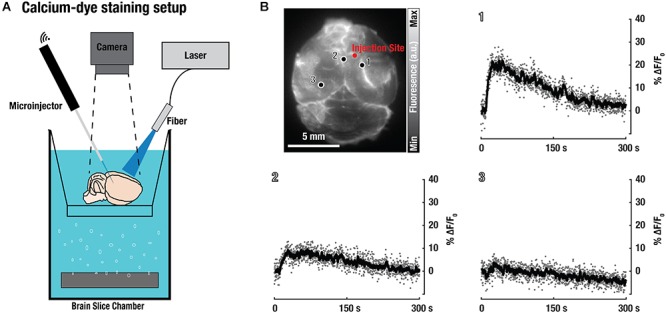
Validation of neuronal functionality based on activity of the calcium-sensitive dye Fluo-3 in the isolated brain preparations. **(A)** Experimental setup used for fluorescence imaging of freshly excised CD-1 mouse brains labeled with the Fluo-3 dye-based calcium indicator. **(B)** Planar (wide-field) fluorescence image of the brain and signal changes in three representative areas show up to 20% increase of the fluorescence intensity following intracortical injection of the epileptic drug pentylenetetrazol.

Additionally, excised brains were labeled with the calcium-indicator dye Fluo-3 in order to validate neuronal functionality (Figure 4). For this, freshly isolated CD-1 mouse brains were stained with Fluo-3-AM and a subsequent intracortical PTZ-injection was used to induce widespread neuronal activity. While only superficial labeling with Fluo-3 was achieved, an increase in calcium-dependent neuronal activity could be observed around the injection site (Figure 4B), thus further demonstrating viability and functionality of the isolated brains.

### Volumetric FONT Imaging

**Figure 5:**
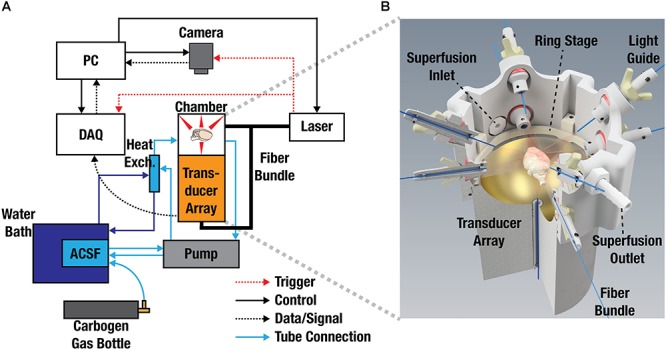
Experimental Layout and system design. **(A)** Layout of the experimental setup used for simultaneous recording of fluorescence and optoacoustic responses from the isolated mouse brains. ACSF, artificial cerebrospinal fluid; DAQ, data acquisition unit; PC, personal computer. **(B)** 3D-rendering of the experimental chamber accommodating the light illumination arrangement, spherical transducer array, superfusion system, and the imaged sample. The isolated brain is placed on top of a transparent polyethylene foil (not shown for simplicity).

A schematic of the imaging setup consisting of a spherical array, 3D-printed superfusion chamber and fluorescence camera is displayed in Figure 5A. To facilitate imaging experiments and provide appropriate experimental and environmental conditions for the isolated brain, a 3D-printed superfusion chamber was developed, as shown in Figure 5B. This allowed for simultaneous planar fluorescence and volumetric optoacoustic imaging of stimulus-evoked brain responses. Nine optical fibers, coupled to an optical parametric oscillator (OPO laser), illuminated the brain from multiple directions to create nearly uniform illumination conditions on its surface. Superfusion inlet and outlet ports were incorporated into the design to establish a physiological environment for the isolated brain by pumping oxygenated ACSF at 37°C around the isolated brain. The spherical ultrasound detection array was designed to provide a field-of-view (FOV) of ∼2 cm^3^ efficiently covering the entire mouse brain with nearly isotropic three-dimensional (3D) resolution of ∼150 μm. This corresponds to about one million individual voxels that can be visualized within the FOV at a volumetric imaging rate of 100 Hz (see section “Materials and Methods” for details of the FONT setup).

**Figure 6:**
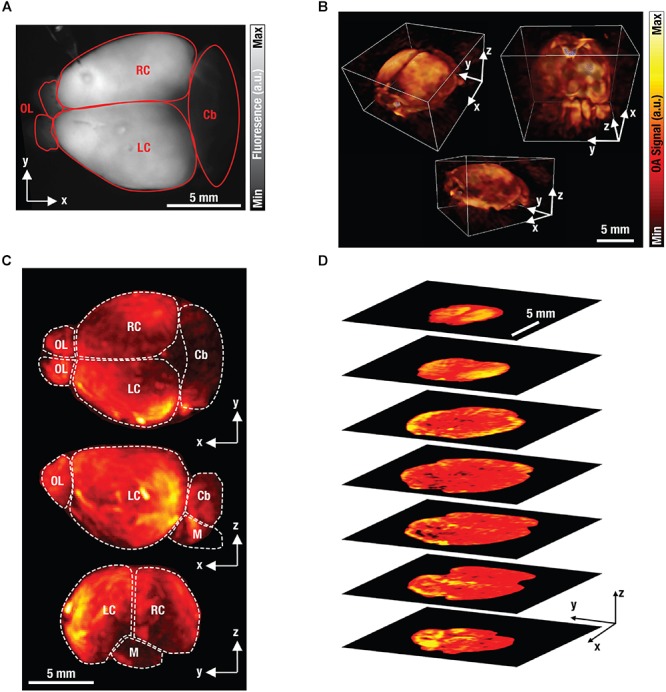
Fluorescence and optoacoustic images of excised brain. **(A)** Planar (wide-field) fluorescence image of an isolated GCaMP6f-labeled mouse brain. For abbreviations see **(C)** below. **(B)** The corresponding volumetric FONT images of the brain. **(C)** Maximum intensity projections of the FONT image. Cb, cerebellum; M, medulla; OL, olfactory bulb; R/LC, right/left cortex. **(D)** Single transverse slices through the brain to highlight the high effective penetration depth of FONT.

Figure 6A shows an isolated GCaMP6f-brain as seen under wide-field fluorescence imaging. Naturally, planar fluorescence can only visualize the brain in 2D along the transverse plane. Whilst some anatomical features can be distinguished, the fluorescence images have a very diffuse appearance making it impossible to discriminate signals originating from different depths due to the intense scattering of light in the brain. As a result, quantification of the measurements is severely compromised since the true origin of the signal cannot be accurately determined. In contrast, FONT is readily capable of imaging the entire volume of the isolated brain along the transverse, sagittal and coronal planes, at a high spatial resolution and in 3D (Figure 6B). A range of anatomical features can clearly be determined, including complete cortices, the cerebellum, the olfactory bulb and the medulla (Figure 6C), thus showcasing the high-resolution volumetric imaging capabilities of FONT and its ability to capture detailed information from the entire isolated brain measuring ∼15 mm along its long axis. Figure 6D further shows single transverse slices of the brain further highlighting the high effective penetration depth of FONT not achievable with other optical imaging modalities.

### Simultaneous Fluorescence and FONT of Stimulated Calcium Activity

**Figure 7:**
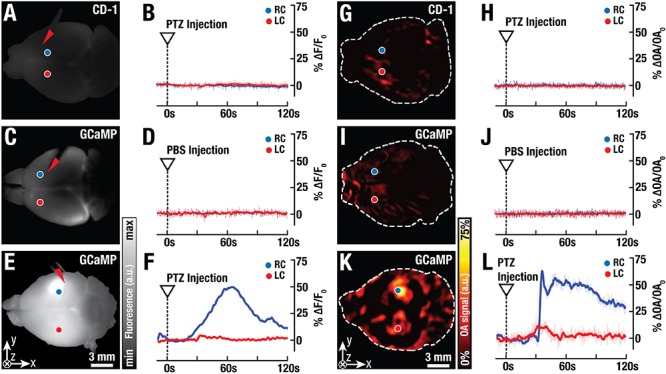
Simultaneously recorded fluorescence and FONT responses in isolated GCaMP6f-brains. **(A)** Planar (wide-field) fluorescence image of an isolated control brain from a CD-1 mouse. **(B)** Normalized time plots from two representative areas of the control brain (20^∗^20 pixels, blue and red circles) reveal no fluorescence activity during injection of the epileptic drug pentylenetetrazol (PTZ) into the brain (injection site indicated by red arrowhead). **(C)** A GCaMP6f-labeled brain exhibits higher background fluorescence levels. **(D)** No activity is detected after injection of phosphate buffered saline (PBS) into the GCaMP6f-labeled brain. **(E)** Planar fluorescence image of a GCaMP6f-labeled brain at ∼40 s post-injection of PTZ. **(F)** The corresponding time trace. **(G)** A 1 mm deep slice of the volumetric FONT image reconstructed from a control CD-1 mouse brain. **(H)** Injection of PTZ into CD-1 mouse brains without GCaMP6f-expression results in no detectable optoacoustic signal variations. **(I)** The FONT slice from a GCaMP6f-expressing brain injected with PBS. **(J)** The corresponding temporal trace reveals no activity. **(K)** Neuronal activation and hence calcium changes are readily observed in the FONT image of GCaMP6f-expressing brains at ∼40 s post PTZ injection. **(L)** Analysis of small volumes of interest in the GCaMP6f-expressing brains (3^∗^3^∗^3 voxels, indicated by blue and red circles) revealed signal increases up to 70% over baseline levels prior to the PTZ injection.

Concurrent fluorescence and FONT imaging of stimulus-evoked calcium activity was used to verify neural activity in the isolated brain model and to validate that signals related to neural calcium dynamics only originate from the GCaMP6f-protein. For this, the neuro-activating agent pentylenetetrazol was injected into the cortex of isolated mouse brains. PTZ is known to interfere with GABAergic signaling thus actuating fast seizure-like activity in the nervous system (Dhir, 2012). Experiments were performed on excised brains from GCaMP6f-expressing mice and CD-1 mice injected with PTZ, with both brains from CD-1 mice injected with PTZ and GCaMP6f-expressing brains injected with PBS, serving as negative controls (Figure 7A,C,G,I). Intracortical injection of PTZ resulted in an increase of fluorescence signals of up to 50% over baseline levels in the GCaMP6f-brains (Figure 7E,F and Supplementary Video S1), while no significant changes could be detected in either of the controls (Figure 7A–D). Notably, the time course of the GCaMP6f fluorescence signal is different from Fluo-3 (Figure 4B), which can be attributed to the different distribution patterns of the two reporters inside the tissue and cells (Dana et al., 2014a; Johnson and Spence, 2010). The molecular reporter Fluo-3 is also more prone to bleaching when compared to protein-based reporters (Gottschalk et al., 2015a). FONT can be used to three-dimensionally map stimulated calcium activity in the whole isolated mouse brain following PTZ injection (Figure 7G,H,K,L). The simultaneously recorded FONT data can directly be compared to the fluorescence results. Injection of PTZ into CD-1 mouse brains (*n* = 3) and injection of PBS into GCaMP6f-brains (*n* = 3) resulted in no significant changes in the optoacoustic signals anywhere inside the brain (Figure 7G–J). On the other hand, neuronal activation by PTZ and hence calcium changes could readily be observed in the GCaMP6f-expressing brains that were stimulated with PTZ (*n* = 4, Figure 7K,L and Supplementary Video S1). Analysis of small volumes of interest (3^∗^3^∗^3 voxels) located 1 mm deep inside the brain again revealed signal increases up to 70% over baseline levels prior to the PTZ injection. Note that the detected OA signal variations were stronger than the corresponding GCaMP6f fluorescence changes, most likely due to the diffuse nature and higher background signal levels of the planar fluorescence modality.

## Discussion and Conclusion

Optoacoustic imaging has recently emerged as a new method for functional brain imaging, enabling non-invasive visualization and quantification of cerebral hemodynamic changes related to functional activity deep in mammalian brains, inaccessible by common high-resolution optical microscopy methods. Yet, strong absorption of light by hemoglobin remains the main limitation for direct visualization of calcium activity in the whole brain. While other blood-free preparations exist, including cell cultures (Pampaloni et al., 2007; Dana et al., 2014b) or brain slices, (Mainen et al., 1999; Llinás et al., 2002) such models fail to represent long-ranging neuronal interactions and network context at a whole brain level. Our study is the first to demonstrate the possibility and validity of a functional excised mouse brain cleared of blood for direct optoacoustic tracking of calcium dynamics associated with neuronal activity in real time in the whole mammalian brain.

In previously reported studies, guinea-pig brains were isolated and perfused through the cortical vasculature, exhibiting a reaction to electrical stimuli for up to 8 h (Mühlethaler et al., 1993). Smaller isolated zebra-fish brains retained functionality for up to 7 days, as verified by the ability to transport horseradish peroxidase and required no perfusion, relying on diffusion to supply the brain with nutrients (Tomizawa et al., 2001). Another study using excised mouse brains also successfully verified recordings of extracellular field potentials and neuronal tracer transport up to 24 h after extraction without the need for vascular perfusion (Von Bohlen and Halbach, 1999). In our own isolated brain preparation, brain viability was validated by Dextran transport experiments while results of the EEG recordings further indicated the presence of neuronal activity for at least 30 min after brain extraction. Since the total imaging experiment duration did not exceed this time, the data acquired in our experiments can be assumed to reflect *in vivo*-like neuronal functionality.

Considering ethical implications, the use of a ketamine/xylazine mixture is an established form of anesthesia, with sedation lasting up to ∼120 min (Erhardt et al., 1984; Gleed and Ludders, 2001). Ketamine produces anesthetic, dissociative, hallucinogenic, amnesic and analgesic effects in the central nervous system by functioning as an NMDAR antagonist (Kohrs and Durieux, 1998; Quibell et al., 2011). In the current study, by employing relatively high ketamine doses and restricting the total experiment duration to 30 min from the start of perfusion, we ensured the excised brain would remain anesthetized throughout the procedure. At the same time, this could affect neuronal transmission functionality by dampening responses to stimuli and the associated calcium fluxes, the extent of which should be addressed in future experiments. Nonetheless, FONT allowed for an unambiguous detection of calcium fluxes as true high resolution 3D-information not affected by intense light scattering in the brain. In contrast, epi-fluorescence recordings failed to provide high resolution maps of depth-resolved calcium dynamics.

In conclusion, we have developed a novel isolated mouse brain preparation and an accompanying imaging setup to optoacoustically monitor calcium dynamics under blood-free conditions. The developed methodology could readily be adapted to work with future generations of far-red- and near-infrared GECIs. Yet, our current results utilizing GCaMP-proteins in the visible range clearly show that deep brain optoacoustic visualization of activity-related calcium signals is achievable in whole rodent brains.

## Materials and Methods

### Isolated Brain Preparation

CD-1 mice aged between 6 and 36 weeks (Envigo, Rossdorf, Germany) were used in these experiments as well as C57BL/6J-Tg(Thy1-GCaMP6f)GP5.5Dkim/J mice aged between 36 and 68 weeks (The Jackson Laboratory, Bar Harbor, ME, United States; stock number 024276) (Dana et al., 2014a). This study was carried out in accordance with the recommendations of the Institute for Biological and Medical Imaging. The protocol was approved by the Government District of Upper Bavaria.

The brain slice chamber (Scientific Products GmbH, Hofheim, Germany) surrounded by ice, was filled with ice-cold oxygenated ACSF and further supplied with freshly oxygenated ACSF (Figure 2A, 3A, 4A). For cardiac perfusion (Figure 1A) ACSF was circulated at a rate of 10 ml/min through a bubble trap and a 25G butterfly infusion set. Mice were anesthetized via a lethal dose of a Ketamine/Xylazine-mixture, administered via an intraperitoneal injection. Surgery commenced once the animal was completely anesthetized as determined by the absence of a toe-pinch reflex at one of the hindpaws. As shown in Figure 1A, surgery began with an incision from the mid abdomen to the sternum. The ventral part of the ribcage was removed to allow unhindered access to the heart. Intracardiac perfusion was carried out by the insertion of a 25G butterfly needle into the left ventricle of the heart followed by an incision into the right atrium. A perfusion pump (Cole-Parmer, Vernon Hills, IL, United States) was used to ensure continuous flow and appropriate pressure of blood and perfusion fluid until both the liver and lungs turned white.

At this stage, decapitation was performed. All tissue and skin surrounding the skull was removed and the skull was rinsed with ice-cold PBS to remove any remaining debris. A cut was then made between the skull and the first cervical vertebra exposing the brain stem (Figure 1B). Using a bone scissors, a second cut was made on both sides of the skull. This cut extended from the foramen magnum to the external auditory meatus, and then from the molar process up to the lachrymal dorsal aspect of the skull. Using forceps, the upper skull plate along with the brain were separated from the lower skull and placed into a Petri dish filled with ice-cold oxygenated ACSF. The brain was separated from the skull using a forceps and placed in a Petri dish filled with fresh ice-cold oxygenated ACSF. Any remaining hair, debris or blood vessels were removed using a fine forceps and pipette. This isolated brain was then directly used for further experiments.

### Dextran Tracing

Axonal tracer transport requires intact, functioning neurons and dextran-amines coupled to fluorescent molecules are known for being transported in both the anterograde and retrograde direction (Vercelli et al., 2000). In order to validate the functionality of axonal transport in the isolated brain preparations, 10 kDa dextran coupled to Texas-red (Thermo Fisher Scientific, Waltham, MA, United States) was injected directly into the cortex of excised brains (Figure 2B,C). For this, freshly isolated brains were placed in a brain slice chamber (Scientific Products GmbH, Hofheim, Germany) filled with ACSF and with constant supply of a mixture of 95% O_2_/5% CO_2_ (Carbogen LAB, The Linde Group, Muenchen, Germany) to keep the solution oxygenated. For intra-brain injection a wireless robotic injection system (Neurostar, Tübingen, Germany) using a 15 to 25 μm diameter pulled glass micro-capillary was utilized. Injections of 0.5 μL volumes were made ∼1 mm deep inside the cortex (*n* = 4). As controls, either PBS or Texas red without Dextran were injected under the same conditions (*n* = 3). Afterward, the brains were fixated in 4% paraformaldehyde either immediately after injection, or after being kept in oxygenated ACSF at 4°C in the dark for 1 h. For evaluation of axonal transport, fixed brains were then sliced into 50 μm-thick sections. For this, the brains were first dehydrated in a solution of 30% sucrose at 4°C for 48 h, to remove water and to prevent ice-crystal formation during cryoslicing. Subsequently, the brains were embedded into an optimal cutting temperature compound (Tissue-Tek^^®^^, Sakura Finetek, Alphen an deen Rijn, Netherlands) and sliced with a CM 1950 Cryo-slicer (Leica Mikrosysteme, Wetzlar, Germany) along the coronal plane. The slices were then mounted onto microscope slides, air-dried for 20 min in the dark and a coverslip was placed on top of the slices and sealed with Vectashield containing DAPI (Vector Laboratories Inc., Burlingame, CA, United States). DAPI stains the DNA and RNA of cells, hence outlining cellular anatomy. Compound brain slice images were captured using an Axio Imager M2 microscope (Carl Zeiss AG, Oberkochen, Germany) fitted with shift-free DAPI and Texas red filter sets (EX TBP 400+495+570, BS FT 410+505+585, EM TBP 460+530+625, Carl Zeiss AG, Oberkochen, Germany). Image acquisition and analysis was done using the Zeiss Zen 2 microscope software.

### Electroencephalography Recording

The experimental setup for Electroencephalography (EEG) recordings is depicted in Figure 3A. EEG-signals were recorded via two custom-made needle electrodes, connected to a DP-311 differential amplifier (Warner Instruments, LLC, Hamden, CT, United States) and the amplified signals were digitized by means of a PowerLab26T data acquisition module (AD Instruments, Sydney, Australia), controlled through a host PC running the Labchart 8 software (AD Instruments, Sydney, Australia). For comparison, *in vivo* EEG-data of CD-1 mice (*n* = 3) was also recorded as described previously (Gottschalk et al., 2017). In order to induce widespread brain activity the epileptic drug PTZ was injected intraperitoneally (IP) (Tang et al., 2015). For *in vivo* EEG-recordings the differential amplifier was set to a high pass of 10 Hz, a low pass of 100 Hz and a gain of 100. After baseline recording, 100 μL of PTZ (100 mg/ml in saline) was injected IP and the mouse was euthanized under anesthesia at the end of the experiment. For recordings in isolated brains (*n* = 3), freshly excised brains were placed in a brain slice chamber (Scientific Products GmbH, Hofheim, Germany) filled with oxygenated ACSF at room temperature (RT). To induce brain activity, 5 μL of PTZ (100 mg/ml in ACSF) was directly injected into the cortex using a pulled glass capillary and a robotic injection system (Neurostar, Tübingen, Germany). For all *ex vivo* EEG-recordings (*n* = 4 for control), the amplifier settings were the same as for the *in vivo* recordings. The recorded EEG signals were processed using MatLab (MathWorks, Natick, United States) to identify periods of elevated brain activity. For this, the EEG spectrogram was calculated as the short-time Fourier transform with a window of 20 s, sufficient for detecting higher frequency components in the 10–20 Hz range corresponding to seizure-like activity caused by PTZ.

### Calcium-Dye Staining

Fluo-3-AM is a fluorescent dye-based calcium indicator that can be used to investigate spatial dynamics of many calcium-dependent signaling processes (Lambert, 2006). Freshly excised CD-1 mouse brains were labeled with Fluo-3 (10 mM Fluo-3-AM and 1 mM Probenecid in oxygenated ACSF) for 20 min in the dark at RT (*n* = 4). After labeling, the brains were washed twice with ACSF, left to rest in oxygenated ACSF for another 15 min in the dark at RT, and were then placed into the experimental setup for fluorescence imaging, as depicted in Figure 4A. A custom-made fiber bundle (CeramOptics GmbH, Bonn, Germany) was used to guide excitation light to the brain from a nanosecond-pulsed optical parametric oscillator laser source (Innolas GmbH, Krailling, Germany). In order to stimulate brain activity, 5 μL of PTZ (100 mg/ml in ACSF) was injected at ∼1 mm depth in the cortex using a pulled glass capillary connected to a robot injection system (Neurostar, Tuebingen, Germany). Fluorescence was recorded during the entire procedure, before and after injection of PTZ.

### Imaging Set-Up

A layout of the imaging set-up used for the simultaneous recording of fluorescence and optoacoustic readings of the excised mouse brain is depicted in Figure 5. Optoacoustic imaging was performed with a custom-made spherical transducer array (Imasonic SaS, Voray, France) with 40 mm radius consisting of 512 piezocomposite elements covering an angle of 140° (1.3π solid angle). The elements have 2.5 mm diameter, 5 MHz central frequency and 100% -6 dB bandwidth, providing an almost isotropic resolution of 150 μm over an approximate FOV of ∼2 cm^3^. The array was held pointing upward via a 3D-printed chamber attached to a X-Y positioning platform (Figure 5B). In the experiments, oxygenated ACSF was pumped through an inlet and an outlet in the holder. ACSF is needed for preserving the brain functional and further provides acoustic coupling for the optoacoustically generated ultrasound waves. The whole chamber was made waterproof by smoothening with a sand paper and applying a silicone finish. Further sealing was ensured with the addition of rubber “O-rings” at all openings including two larger “O-rings” surrounding the transducer array. The excised brain was placed approximately at the center of the FOV of the spherical matrix transducer array lying upon a ∼10 μm transparent polyethylene foil glued to a 3D-printed ring stage. Brain illumination was done via a self-developed fiber bundle consisting of nine fibers with a core diameter of 600 μm. One of the fibers was inserted into a cylindrical cavity of the spherical array to illuminate the bottom part of the brain while the other 8 were embedded in apertures in the array holder equally spaced at 90° in azimuth and with polar angles of 5.7° and 37° (Figure 5B). The illumination source was an optical parametric oscillator (OPO)-based laser (Innolas GmbH, Krailling, Germany) providing short (<10 ns) pulses at 25 Hz with optical wavelength set to 488 nm. The 512 optoacoustic signals detected by the array elements were simultaneously digitized with a custom-made data acquisition system (Falkenstein Microsystem GmbH, Taufkirchen, Germany) triggered with the Q-switch output of the laser. The digitized signals were transferred to a PC via 1 Gbit/s Ethernet for further processing. A high speed EMCCD camera (Andor Technology Ltd., Belfast, United Kingdom) located on top of the chamber (pointing downward) was used to record the fluorescence responses of the isolated brains being illuminated with the laser source. The camera was equipped with a 105 mm Nikon F mount objective (Nikon, Chiyoda, Tokio, Japan) and a one-inch emission filter with 525 nm center wavelength and 39 nm bandwidth (MF525-39, Thorlabs Inc., Newton, United States). The acquisition time of the camera was set to 200 ms, corresponding to the integration of five laser pulses. The relative positions of the transducer array and the camera were manually adjusted with the X-Y positioning platform holding the transducer array.

### Data Analysis and Processing

The relative fluorescence signal changes (ΔF/F_0_) were calculated from the recorded images as changes in signal intensity following the PTZ injection. These changes correspond to the calcium responses evoked by neural activity. The baseline fluorescence signal level F_0_ was calculated as the average of frames over 5 s preceding the injection. Volumetric (3D) optoacoustic images were reconstructed from the acquired signals using a graphics processing unit based implementation of a back-projection formula (Dean-Ben et al., 2013). Before reconstruction, the signals were deconvolved with the impulse response of the transducer array elements and a band-pass filter with cut-off frequencies of 0.1 and 6 MHz was applied. The reconstruction of the optoacoustic data was performed on a grid of 150 × 150 × 100 voxels^3^ (equivalent to 15 × 15 × 10 mm^3^) to match the spatial resolution of the system.

## Author Contributions

OD performed all experiments. OD, BML, and JR designed and fabricated the 3D-printed chamber. SG, OD, BML, JR, and XLDB analyzed and processed the data. SG, SS, and DR validated the data analysis. SG, SS, and DR designed and led the study. All authors wrote, read, and accepted the manuscript.

## Conflict of Interest Statement

The authors declare that the research was conducted in the absence of any commercial or financial relationships that could be construed as a potential conflict of interest.

## Footnotes

1 http://connectivity.brain-map.org/

## Supplementary Material

VIDEO S1 Video of a concurrent fluorescence and optoacoustic recording of a GCaMP6f-brain. The video shows the changes in fluorescence (planar image) and the optoacoustic signal (MIP, maximum intensity projection) upon injection of the epileptic drug pentylenetetrazol (PTZ, injection at time 0). Clear neuronal activation by PTZ as reflected by calcium changes can be observed in both fluorescence and optoacoustic data. CB, cerebellum; L/RC, left/right cortex; OL, olfactory bulb.
